# Meningeal myofibroblastoma in the frontal lobe: A case report

**DOI:** 10.3892/ol.2014.2290

**Published:** 2014-06-26

**Authors:** QINGSHENG XU, YIPING FENG, PAN WU, YONGQING ZHOU

**Affiliations:** Department of Neurosurgery, The First Affiliated Hospital, College of Medicine, Zhejiang University, Hangzhou, Zhejiang, P.R. China

**Keywords:** myofibroblastoma, meningeal neoplasm

## Abstract

Myofibroblastoma is a benign tumor composed of spindle cells in clusters and fascicles. To date, only three cases of intracranial myofibroblastoma have been reported. The present study reports the case of a 47-year-old female with meningeal myofibroblastoma. The patient had a history of ovarian cyst resection and presented with paroxysmal mild headaches that had been apparent for 4 years. Magnetic resonance imaging disclosed a well-circumscribed mass in the left frontal lobe. A resection of the mass was performed. Abundant fascicular clusters of spindle- and oval-shaped cells were found by conventional histopathology. Immunohistochemical staining demonstrated that these cells were strongly positive for smooth muscle actin, weakly positive for epithelial membrane antigen and negative for cluster of differentiation (CD)117, CD34, S-100 or desmin, with a Ki-67 index of >10%. These results supported the diagnosis of myofibroblastoma. No recurrence of the mass was found during the 24-month follow-up period. Overall, the patient exhibited a rare type of meningeal neoplasm. Resection of the tumor proved to be successful and no recurrence were found. Histopathological and immunohistochemical staining is crucial to form a diagnosis. To the best of our knowledge, the present study is the first to show the presence of myofibroblastoma in the left frontal lobe.

## Introduction

Myofibroblastoma is a benign and rare mesenchymal neoplasm composed of spindle cells in clusters and fascicles, with interspersed bands of hyalinized collagen (1,2). The majority of the masses are located in the breast, however, the number of extramammary myofibroblastoma cases being reported is increasing (2–6). Only three cases of intracranial myofibroblastoma have been reported (7–9). Histopathological and immunohistochemical staining is crucial to determine a diagnosis of intracranial myofibroblastoma. Tumor resectioning may be a useful treatment. The present study describes the case of a female patient with meningeal myofibroblastoma. In this report we describe the clinical and pathological features of this rare tumor and discuss the differential diagnoses. To the best of our knowledge, this is the first study to show the presence of myofibroblastoma in the left frontal lobe. Patient provided written informed consent.

## Case report

**Figure 1:**
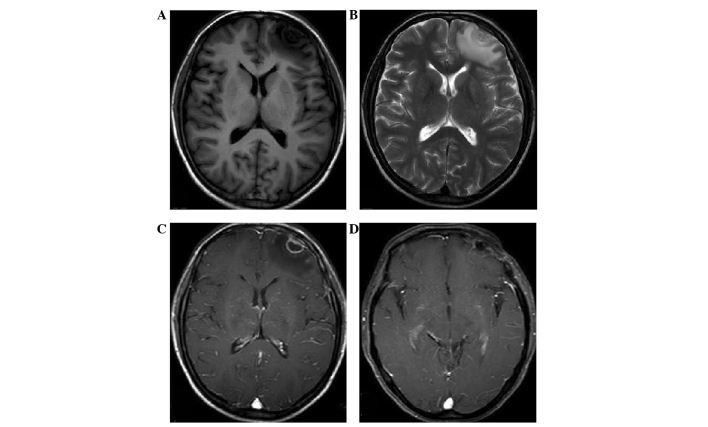
MRI scans disclosing a well-circumscribed mass in the left frontal lobe. (A) The mass appears as hypointense on T1-weighted images and (B) was of mixed intensity on T2-weighted images. (C) Gadolinium-diethylene triamine pentaacetic acid-enhanced T1-weighted images demonstrating the ring-like enhanced boundary of the mass. (D) No tumor recurrence at the 24-month follow-up. MRI, magnetic resonance imaging.

A 47-year-old female with a history of ovarian cyst resection presented to The First Affiliated Hospital (College of Medicine, Zheijian University, Hangzhou, Zhejiang, China) with paroxysmal mild headaches that had been apparent for 4 years. The headaches had increased in intensity over the past 6 days. Computed tomography (CT) revealed a low-density mass lesion in the frontal lobe. The physical examination was normal. The analysis of tumor markers showed no abnormal findings. A cerebro-spinal fluid examination disclosed a slightly elevated level of protein at 0.50 g/l (normal, 0.15–0.45 g/l), with no other abnormal findings. Magnetic resonance imaging (MRI) disclosed a well-circumscribed mass in the left frontal lobe (Fig. 1). The clear boundary and surrounding extruded brain tissue indicated that the mass had undergone expansive growth. The mass appeared as hypointense on T1-weighted images and was of mixed intensity on T2-weighted images (Fig. 1A and B). Gadolinium-diethylene triamine pentaacetic acid (DTPA)-enhanced T1-weighted images revealed that the mass was heterogeneously enhanced, with ring-like enhancement of the boundary. The cerebral dura on the base of the lesion was also believed to be contrast-enhanced, with thickening of the left frontal bone, which may have been a result of reactive hypervascularity or tumoral invasion of the dura (Fig. 1C). The preliminary diagnosis of the mass was of a meningioma.

A resection of the mass was performed; the mass appeared to be dusty pink color and had a resilient structure, with abundant blood supply. Significant adhesion to the surrounding brain tissue was present, together with severe edema surrounding the mass. The base of the mass was attached to the convex dura, with focal localization on the dura of the frontal basal section. Diagnosis of the frozen section performed at the time of surgery disclosed a suspected myofibroblastoma. The patient’s headache had resolved at 2 weeks post-surgery. CT revealed postoperative manifestation and no evidence of lesion recurrence. Anti-epilepsy therapy was recommended when the patient was discharged from the hospital. The patient recovered well, with no evidence of mass recurrence on MRI to date at 24-months post-surgery (Fig. 1D).

**Figure 2:**
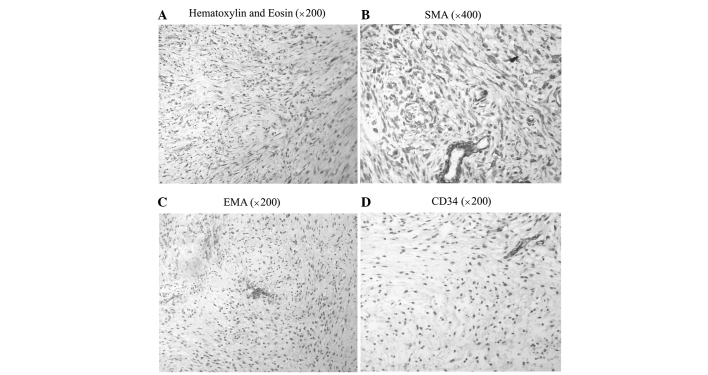
(A) Fascicular clusters of spindle- and oval-shaped cells arranged in interlaced or swirled patterns (hematoxylin and eosin staining). (B) Strongly positive staining of the cells for smooth muscle actin (SMA). (C) Focally positive staining of the cells for epithelial membrane antigen (EMA). (D) Staining of the tumor cells for cluster of differentiation (CD)34 is negative.

The histopathological findings revealed abundant fascicular clusters of spindle- and oval-shaped cells, which were arranged in interlaced or swirled patterns. Elongated to oval-shaped nuclei with inconspicuous nucleoli were another distinguishing feature. Finely-dispersed chromatin and poorly-defined cytoplasm were also observed. Mitotic figures were rare. Mucinous degeneration and necrosis were observed in the eosinophilic area, while an occasional lymphocyte and neutrophil were seen between tumor cells (Fig. 2A).

Immunohistochemical staining demonstrated that the majority of the tumor cells were strongly positive for smooth muscle actin (SMA; Fig. 2B), the Ki-67 index was >10% and only a few cells were positive for epithelial membrane antigen (EMA; Fig. 2C). However, the tumor cells were negative for cluster of differentiation (CD)117, CD34 (Fig. 2C), S-100 and desmin. All of the pathological evidence supported the diagnosis of myofibroblastoma.

## Discussion

Studies on myofibroblastoma in the central nervous system are extremely rare. To the best of our knowledge, only three cases of intracranial myofibroblastoma have previously been reported (7–9). The details of these case studies are summarized in Table I. From these data, there is a trend towards females being more likely to suffer from intracranial myofibroblastoma. The age in the studies varies between 9 and 70 years. All intracranial myofibroblastomas have definite or suspected attachment to the dura.

Myofibroblastoma is a well-circumscribed benign tumor. Histopathological findings demonstrate that the tumor is composed of spindle cells in clusters and fascicles, with thick interspersed hyalinized collagen bands. It has features of fibroblasts and smooth muscle cells, with frequent mitoses. The cells are also characterized by elongated to oval-shaped nuclei, irregular nuclear contours, finely-dispersed chromatin and poorly-defined cytoplasm (1,2,7,9,10). Immunohistochemical staining has shown that the tumor cells are strongly positive for SMA and vimentin and weakly positive for desmin and CD34 (8,9). However, the reactivity to factor VIII-related antigen (a marker of endothelial cells), EMA, MAK-6 (cytokeratin; a marker of epithelial cells and meningeal cells) and glial fibrillary acidic protein are negative (8). Ultrastructural examination discloses that the mass is composed of myofibroblastic cells and fibroblastic cells. The cytoplasm of the myofibroblastic cells contains abundant dilated rough endoplasmic reticulum (rER), while the cytoplasm of the fibroblastic cells contains actin-like microfilaments, with dense bodies, and abundant rER (9).

The clinical manifestations of intracranial myofibroblastoma are similar to meningioma and include intracranial hypertension, skull destruction and the presence of systematic symptoms. Headaches caused by intracranial hypertension subsequent to the effects of a mass are extremely common. The masses may grow slowly, as all patients tend to have a long medical history prior to their admittance to hospital. Intratumoral hemorrhage may also be a feature of the mass (9).

CT and MRI are useful imaging methods in diagnosing myofibroblastoma, as it is well-circumscribed on each of these techniques. The mass can appear as a low- or mixed low- and high-density mass on CT (9). In the present study, the mass was hypointense on T1-weighted images and was of mixed intensity on T2-weighted images (Fig. 1A and B). This result is different to that in the study by Shinojima *et al* (9), which showed that the mass was isointense on T1- and hypointense on T2-weighted MI. This difference may be due to intratumoral hemorrhage in the previous case. The mass showed heterogeneous contrast enhancement on gadolinium-DTPA-enhanced T1-weighted images in the present study; this has also been demonstrated in the two previous cases (8,9). One notable point was that the cerebral dura on the base of the lesion was also contrast-enhanced, with thickening of the left frontal bone in the present patient. This is similar to the dural tail sign, which indicates that it may be a result of the invasion of dural vessels by tumor cells and packing at the point of tumor attachment, reactive hypervascularity or tumoral invasion of the dura (11). The ring-like enhanced boundary in the current patient was mostly likely the meninges, due to the continuity between the boundary and the meninges. All the myofibroblastomas in the previous three cases, plus that in the present study, had definite or suspected attachments to the dura, which indicated their origination from the meninges, possibly from modified fibroblasts or pre-existing myofibroblasts (9).

The differential diagnosis of meningeal myofibroblastoma includes other spindle cell neoplasms of the meninges, such as solitary fibrous tumors (SFTs), fibrous meningiomas and hemangiopericytomas.

SFTs are rare tumors that can also occur in the meninges. Histopathological findings demonstrate the presence of numerous monomorphic spindle- or oval-shaped cells and diffuse intercellular reticulin fibers. These findings are similar to those of myofibroblastoma. However, unlike for myofibroblastoma, branching hemangiopericytoma-like vessels and rare mitotic figures are characteristics that are also present (12,13). Immunohistochemical analysis has shown that SFTs are strongly and diffusely positive for CD34, vimentin, B-cell lymphoma-2 and CD99, but negative for SMA, EMA or S-100 protein (14–17). No cells with the features of smooth muscle cells are found under ultrastructural examination (15).

Fibrous meningioma is another type of meningioma. Unlike myofibroblastoma, fibrous meningiomas are glycogen-containing tumors. Additionally, a storiform pattern, psammoma body and collagen calcification are defining characteristics (12,18). Immunohistochemical analysis shows that these tumors are positive for vimentin (100%), focal EMA (80%), S-100 protein (80%) and collagen IV (25%). CD34 staining is patchy and weak (60%) (18,19).

Meningeal hemangiopericytomas (HPCs) are also meningeal neoplasms, and are composed of oval- to spindle-shaped cells, with dense intercellular reticulin fibers. However, unlike myofibroblastomas, HPCs are prone to multiple recurrences and eventual metastasis. Another difference can be found in the existence of numerous small blood vessels (12,19,20). HPC is characteristically positive for vimentin, factor XIIIa and Leu-7, and CD34 staining is patchy and weakly positive. Focal desmin and cytokeratin positivity is occasional, with negative EMA and S-100 staining (12,19,21). On CT and MRI, the tumors are characterized by irregular borders rather than the well-defined borders of a myofibroblastoma (20).

The prognosis of meningeal myofibroblastoma is optimistic. The masses are slow-growing and the histopathology findings show no evidence of malignancy. In the present patient, the resection of the tumor proved to be a successful treatment and no recurrence was found, similar to the two previous cases (7,9).

The present patient exhibited a rare type of meningeal neoplasm. Histopathological and immunohistochemical staining is crucial to identify the diagnosis of a myofibroblastoma. Further study is required to identify the origin of the tumor and the association between the tumor and other meningeal neoplasms, such as SFT.

## Figures and Tables

**Table I:** Information on reported intracranial myofibroblastoma cases.

First author, year (ref.)	Age, years	Symptoms	Location of the lesion	Size, cm
Carneiro *et al,* 1989 (3)	9	Diplopia, convergent strabismus	Meninges overlying the parietal lobe	1.2
		right ocular protrusion	No data	3.5
Prayson *et al,* 1993 (4)	70	Headache, visual change	Posterior falx below the sagittal sinus	2×3×4
			right occipital lobe	2×4×6
			Adjacent to the superior sagittal sinus	No data
Shinojima *et al,* 2002 (5)	34	Headache, visual disturbance	Suprasellar region	2.5×2×3
Present study	47	Headache	Frontal lobe	1.7×1.5×2

## Abbreviations

MRI: magnetic resonance imaging
CT: computed tomography
CD: cluster of differentiation
SMA: smooth muscle actin
EMA: epithelial membrane antigen
DTPA: diethylene triamine pentaacetic acid
rER: rough endoplasmic reticulum
SFT: solitary fibrous tumor
HPC: hemangiopericytoma
